# Supplementary material for: Impacts of climate change on the distribution pattern of Himalayan Rhubarb (Rheum australe D. Don) in Nepal Himalaya
Source: PLoS One. 2025 May 28;20(5):e0323755. doi: 10.1371/journal.pone.0323755 (PMC12118860; doi:10.1371/journal.pone.0323755)
Supplement: Attachment — Submitted filename: Response to reviewers.docx [file pone.0323755.s003.docx]

Dear Daniel de Paiva Silva, Ph.D.
Academic Editor
PLOS ONE,

Thank you for providing an opportunity to revise the manuscript “PONE-D-24-47340 Impacts of climate change on the distribution pattern of Himalayan Rhubarb (Rheum australe D.Don) in Nepal Himalaya”. We have thoroughly revised the manuscript based on the suggestions/comments of the reviewers. Indeed, the comments/suggestions given by the reviewers helped us immensely to improve the clarity of the manuscript. Below, we provide the point-by-point responses to the editorial and reviewers’ comments. We now believe that the revised manuscript is suitable for publication in PLOS ONE.

Sincerely yours

Babu Ram Paudel

On behalf of all authors

******************************

Dear Dr. Paudel,

Thank you for submitting your manuscript to PLOS ONE. After careful consideration, we feel that it has merit but does not fully meet PLOS ONE’s publication criteria as it currently stands. Therefore, we invite you to submit a revised version of the manuscript that addresses the points raised during the review process……………………..

We look forward to receiving your revised manuscript.

Kind regards,

Daniel de Paiva Silva, Ph.D.
Academic Editor
PLOS ONE

Comments of the Editorial office

Journal Requirements:

When submitting your revision, we need you to address these additional requirements.

1. Please ensure that your manuscript meets PLOS ONE's style requirements, including those for file naming. The PLOS ONE style templates can be found at

<https://journals.plos.org/plosone/s/file?id=wjVg/PLOSOne_formatting_sample_main_body.pdf> and

<https://journals.plos.org/plosone/s/file?id=ba62/PLOSOne_formatting_sample_title_authors_affiliations.pdf>

2. Thank you for stating the following financial disclosure:

“The study was supported by an innovative research grant (Grant Number: TU-NPAR-078/79-4-04) from the Research Directorate, Office of the Rector, Tribhuvan University, Nepal.”

Please state what role the funders took in the study.  If the funders had no role, please state: "The funders had no role in study design, data collection and analysis, decision to publish, or preparation of the manuscript."

If this statement is not correct you must amend it as needed.

Please include this amended Role of Funder statement in your cover letter; we will change the online submission form on your behalf.

Re: The financial disclosure statement has been amended to reflect the suggested changes which now appears as “The study was supported by an innovative research grant (Grant Number: TU-NPAR-078/79-4-04) from the Research Directorate, Office of the Rector, Tribhuvan University, Nepal. The funders had no role in study design, data collection and analysis, the decision to publish, or preparation of the manuscript”

3. We note that Figures 1, 4, 5 and 6 in your submission contain [map/satellite] images which may be copyrighted. All PLOS content is published under the Creative Commons Attribution License (CC BY 4.0), which means that the manuscript, images, and Supporting Information files will be freely available online, and any third party is permitted to access, download, copy, distribute, and use these materials in any way, even commercially, with proper attribution. For these reasons, we cannot publish previously copyrighted maps or satellite images created using proprietary data, such as Google software (Google Maps, Street View, and Earth). For more information, see our copyright guidelines: <http://journals.plos.org/plosone/s/licenses-and-copyright>.

We require you to either (1) present written permission from the copyright holder to publish these figures specifically under the CC BY 4.0 license, or (2) remove the figures from your submission:

     1. You may seek permission from the original copyright holder of Figures 2, 3, 5 and 6  to publish the content specifically under the CC BY 4.0 license.

We recommend that you contact the original copyright holder with the Content Permission Form (http://journals.plos.org/plosone/s/file?id=7c09/content-permission-form.pdf) and the following text:

“I request permission for the open-access journal PLOS ONE to publish XXX under the Creative Commons Attribution License (CCAL) CC BY 4.0 (http://creativecommons.org/licenses/by/4.0/). Please be aware that this license allows unrestricted use and distribution, even commercially, by third parties. Please reply and provide explicit written permission to publish XXX under a CC BY license and complete the attached form.”

Please upload the completed Content Permission Form or other proof of granted permissions as an "Other" file with your submission.

In the figure caption of the copyrighted figure, please include the following text: “Reprinted from [ref] under a CC BY license, with permission from [name of publisher], original copyright [original copyright year].”

   2. If you are unable to obtain permission from the original copyright holder to publish these figures under the CC BY 4.0 license or if the copyright holder’s requirements are incompatible with the CC BY 4.0 license, please either i) remove the figure or ii) supply a replacement figure that complies with the CC BY 4.0 license. Please check copyright information on all replacement figures and update the figure caption with source information. If applicable, please specify in the figure caption text when a figure is similar but not identical to the original image and is therefore for illustrative purposes only.

The following resources for replacing copyrighted map figures may be helpful:

USGS National Map Viewer (public domain): http://viewer.nationalmap.gov/viewer/

The Gateway to Astronaut Photography of Earth (public domain): <http://eol.jsc.nasa.gov/sseop/clickmap/>

Maps at the CIA (public domain): https://www.cia.gov/library/publications/the-world-factbook/index.html and <https://www.cia.gov/library/publications/cia-maps-publications/index.html>

NASA Earth Observatory (public domain): http://earthobservatory.nasa.gov/

Landsat: <http://landsat.visibleearth.nasa.gov/>

USGS EROS (Earth Resources Observatory and Science (EROS) Center) (public domain): [http://eros.usgs.gov/#](http://eros.usgs.gov/)

Natural Earth (public domain): <http://www.naturalearthdata.com/>

Re: All the maps used here are the Maxent-generated outputs of the bioclimatic data downloaded from the public domain “ <https://www.worldclim.org/data/worldclim21.html>’ which has also been duly cited in the manuscript. Therefore, we believe that there are no copyright issues for the map.

Editor’s Comments:

Dear Dr. Paudel,
After this first review round you have received a major and a minor reviews. Therefore, your manuscript may be accepted for publication in PLoS One after a major review is considered. Please take careful consideration on the suggestions provided by both reviewers. Considering the extent of reviews you need to do, I believe you will need a three-month period to complete the changes required by the reviewers. Please do not forget to prepare a rebuttal letter, showing the changes you performed following the reviewers' suggestions and those that you did not agree with, informing your reviewers the reason why the changes were not performed.

Sincerely,
Daniel Silva

Re: Thank you for providing an opportunity to revise the manuscript. We have thoroughly revised the manuscript based on the suggestions/comments of the reviewers. Below, we provide the point-by-point responses to the editorial and reviewers’ comments. The line numbers in the response letter refers to the line number line number in the revised clean version of the manuscript.

Reviewers' comments:

Reviewer's Responses to Questions

**Comments to the Author**

1. Is the manuscript technically sound, and do the data support the conclusions?

The manuscript must describe a technically sound piece of scientific research with data that supports the conclusions. Experiments must have been conducted rigorously, with appropriate controls, replication, and sample sizes. The conclusions must be drawn appropriately based on the data presented.

Reviewer #1: Partly

Reviewer #2: Yes

2. Has the statistical analysis been performed appropriately and rigorously?

Reviewer #1: Yes

Reviewer #2: Yes

3. Have the authors made all data underlying the findings in their manuscript fully available?

The [PLOS Data policy](http://www.plosone.org/static/policies.action#sharing) requires authors to make all data underlying the findings described in their manuscript fully available without restriction, with rare exception (please refer to the Data Availability Statement in the manuscript PDF file). The data should be provided as part of the manuscript or its supporting information or deposited to a public repository. For example, in addition to summary statistics, the data points behind means, medians and variance measures should be available. If there are restrictions on publicly sharing data—e.g. participant privacy or use of data from a third party—those must be specified.

Reviewer #1: Yes

Reviewer #2: Yes

4. Is the manuscript presented in an intelligible fashion and written in standard English?

PLOS ONE does not copyedit accepted manuscripts, so the language in submitted articles must be clear, correct, and unambiguous. Any typographical or grammatical errors should be corrected at revision, so please note any specific errors here.

Reviewer #1: Yes

Reviewer #2: Yes

5. Review Comments to the Author

Please use the space provided to explain your answers to the questions above. You may also include additional comments for the author, including concerns about dual publication, research ethics, or publication ethics. (Please upload your review as an attachment if it exceeds 20,000 characters)

Reviewer #1

 Here are my suggestions: Depth in Discussion of Climate Impacts: While mentioning the general effects of climate change, the introduction could explore in more detail how these impacts affect Nepal in particular (would CO2 emissions influence plant distribution/suitability??? Would rainfall and temperature alter photosynthetic rate???), with an emphasis on interactions between environmental and socioeconomic factors.... It is interesting to clarify what the effect of climate change is.

Re: Thank you for your suggestions. We agree. …CO2 emissions along with other variables (rainfall, temp, soil nutrient/types may influence plant photosynthesis, growth, and distribution. These issues are addressed in the introduction in the light of available literature. Please refer to the line numbers 32-55 in the introduction and line numbers 619-629 in the discussion section. We believe that our prediction models under different climate scenarios characterized by various concentrations of carbon dioxide emissions serve as the best proxy to assess the role of increased carbon dioxide concentrations/temperature on the distribution of our study species. We discuss the implication of the result under different emission scenarios in the discussion section at line number 679-691. However, as we lack empirical data on the impact of increased carbon dioxide concentration/ temperature on the physiology and distribution of Nepalese plant species, we were unable to explicitly include these variables in the models (which is indeed not in practice by SDM studies).

We believe the current revision satisfies the main concern of the reviewer. We would be pleased to revise it again (if necessary) if the reviewer could kindly share with us some literature that might be helpful for us.

Part of the introduction could be moved to the discussion, as it contains a critical analysis of previous studies, methodological limitations, and implications for future research—typical elements of a discussion section. Specifically, the following points would be more appropriate in the discussion: 1. **Model and Data Comparison**: An assessment of the limitations of CMIP5 data and a rationale for using CMIP6 data, including specific benefits in terms of reduced lifespans and improved predictions in climate forecasts (lines 85–94). And lines 95-108... This excerpt is a critical evaluation of previous studies, highlighting limitations and justifying the methodological and conceptual advances of the current study. As such, it would be more appropriate in the discussion section of the article, preferably in a paragraph that analyzes how the results found compare with the existing literature.

Re: We have reworded and moved these sentences to the beginning of the discussion section. Please refer to the line numbers 500-554 in the revised (clean) version of the manuscript.

Lines 60-83, in the excerpt provided, the author does not clearly specify whether he used SDM (Species Distribution Modeling) or ENM (Ecological Niche Modeling) in the study. He mentions both terms interchangeably, but the focus seems to be on modeling the distribution of the species based on occurrence data and environmental variables, which is characteristic of both SDM and ENM.
...MaxEnt is often associated with species distribution modeling (SDM). However, the methodology can also be considered within the context of ENM, since it involves the use of environmental variables to estimate the ecological niche of the species. Therefore, the author does not make an explicit distinction... Make it clear to the reader what was done.

Re: We performed species distribution modelling for our study species. We have dropped the terminology “ENM (Ecological Niche Modeling)” from the manuscript to avoid confusion for the readers.
In the Natural Distribution Data Section: mention the date of access to the databases.

Re: The occurrence records of the species from the online databases were accessed on 20 June 2024. We have corrected the manuscript accordingly. Please refer to line numbers 138-139.
Environmental Variables: it is necessary to justify the choice for the use of each variable. The reader needs to understand the role of temperature and precipitation in the species.

Re: We considered 24 environmental variables comprising the combination of 19 bioclimatic variables and 5 topographic factors (table 1) as the predictors of habitat suitability of our study species because several previous studies have suggested the important role of these environmental variables for the distribution of plant species in mountain regions such as Nepal. We have now added this information to the main manuscript. Please refer to the line numbers 147-195. However, due to the collinearity problem that causes overprediction affecting the accuracy of the predictions, we eliminated the collinear variables in the final model development. After the elimination of the collinear variables, 11 variables were retained which were used in the final model. This has been clearly stated in the main manuscript. Please refer to the line numbers 147-195.
Why was the variable solar radiation not considered? Solar radiation is essential for plant growth and development, as it is the source of energy for photosynthesis.

Re: Thank you for this suggestion. Besides the above 24 variables, we attempted to include other variables including solar radiation, human footprint index, and soil PH. However, those data were missing at several distribution points of our study system. So, we were unable to include these important variables in the model development.
Results: Improve the explanation of the criteria used for the classification of habitat zones and the organization of data in the tables.

Re: The suitable habitat zones were classified into three different categories: low suitable (Threshold value ≤ 0.4, i.e., up to 40% probability of occurrence), medium suitable (Threshold value between 0.4 to 0.6), and high suitable (Threshold value > 0.6) following the criteria used by Rawat et al 2022 and Yang et al 2013. We corrected the manuscript accordingly. Please refer to the line numbers 260-264.

For the data arrangement in the tables…

For table 1: 19 bioclimatic variables were kept at first and then additional environmental variables were arranged in alphabetical order. The table has been corrected accordingly.

For Table 2: The environmental variables in the table are arranged in the order (high to low) of their contribution to the model development. This explanation has been added to the table legend.

For Tables 3 and 5: We think, no specific explanation is required.

For Table 4: The districts in the table are arranged in the order of high to low suitable areas of R. australe for the current scenario. This explanation has been added to the table legend.

For Table 6: The districts in the table are arranged in the order of high to low projected loss of suitable habitat of R. australe for the climate scenario SSP126 for the period 2021-2040. This explanation has been added to the table legend.

The figures and graphs mentioned in the article (such as Figures 4-6) are essential to illustrate the projections, but the details on how each figure contributes to the understanding of the results could be more explanatory. This includes the description of what each colored area represents and how it relates to the zones of suitable habitat in each scenario.

Re: Description added in the figure legend to reflect the changes suggested.

Appreciation of AUC and TSS Values: Although the AUC (0.921) and TSS (0.68) values are mentioned as indicators of model accuracy, a clearer interpretation of these numbers would be useful. For example, it could be detailed what these results imply for the reliability of the projections in different climate scenarios. It would also be interesting to discuss the impact of a TSS of 0.68 in terms of model predictability and compare it with other similar studies.

Re: We have added the description for the AUC and TSS values in terms of model predictability. Please refer to line number 231-244 in the method section. We also discussed the model's applicability under the similar TSS values range (TSS > 0.6) with reference to other similar studies. Please refer to the line numbers 314-324

In the discussion:
I suggest removing the subtitles, the discussion section becomes more fluid and less segmented, which makes it easier to understand the text as a whole.

Re: As suggested, we have removed the subtitle from the discussion.
Deeper exploration of limitations: Although the discussion mentions methodological advances, a more detailed analysis of the limitations of the study could be useful. For example, the accuracy of species occurrence data and the challenges associated with the use of climate models (such as uncertainty in climate scenarios) could be better explored.

Re: We have revisited the limitations of the current study including all the points you suggested. However, to address the concerns of reviewer 2, we have softened the words. Please refer to line numbers 500-554.

Expanding on comparisons with other studies: Although the discussion briefly mentions the contributions of the study to the existing literature, it would be interesting to see a more in-depth comparison with other similar studies on the distribution of medicinal plants in mountainous regions or the impact of climate change on other species. This would help to better contextualize the findings.

Re: Done. Please refer to the line numbers 563-576 and 595-598.
Clarity on the implications of the results for the future: The text could provide more detail on how the results may impact the management and conservation of R. australe in the long term. It would be interesting to expand on the policy recommendations, considering different climate change scenarios and how they could affect cultivation and conservation areas.
Re: We have clarified the implications of the result about different future climate scenarios. Please refer to line numbers 692-719.

Reviewer #2

Thank you for the opportunity to learn from your work.

This is an interesting paper that contributes to the debate about the effects of climate change over economically valuable species. However, the Discussion needs to be revised to make sense in a journal like Plos One. The results will be better explored if the ecological and economic impacts of the extinction of Rheum australe are addressed in greater depth and in a broader way, moving away from the logic of dialogue between similar articles.

Re: The ecological and economic implications are briefly discussed in the revised manuscript. Please refer to line numbers 652-678. However, due to the lack of sufficient data, we are unable to explicitly discuss this aspect.

We believe the current revision satisfies the main concern of the reviewer. We would be pleased to revise it again (if necessary) if the reviewer could kindly share with us some literature that might be helpful for us.

I'm sending my considerations line by line below.

Line 30: Those keywords are already on title; please replace them.

Re: Thank you for this valuable suggestion. We have corrected the keywords
Line 83: It is also important to talk about the limitations of the method (Maxent) somewhere in the text

Re: We have discussed the limitation of the study including the limitation of the Maxent model and our efforts to minimize the limitations in the discussion section and also in the method section. Please refer to the line number at 214-229 (method section) and 502-506, and 521-553 (discussion section).

Line 147: Fig 1. Please, improve the plant image resolution.

Re: We have replaced the image.

Line 284: Please italicize “Rheum australe”.

Re: Done. Now at line 269.
Line 296: Consider excluding terms that can be seen as judgment value, as “our ’extensive’ observation”, “actual high”.

Re: We have softened the wording in the manuscript and those words no longer appear in the revised manuscript.
Lines 307-310: But wouldn't it be possible for this environment to also be suitable? What if the species just didn't make it there? My point is, given that the model indicates these areas as suitable, and how severe climate change could be, aren't you being too conservative in considering only forests and ragelands as suitable areas? I'd like to hear your thoughts on this.

Re: We agree with the reviewer that areas predicted suitable by the model other than forests/rangelands could also be likely developed as future plantation sites for *Rheum australe*. However, our long-term observation and habitat assessment of *Rheum australe* across several populations in the Nepalese mountains reveals that *R. australe* is found only in the forest or rangelands, and not in other land use types. Moreover, current distribution records also support our observation. Therefore, we expect that the prediction would be more precise if we include only the forests and range lands as the suitable habitats of the study species. We have addressed this issue in the revised manuscript also. Please refer to line number 281-285. We hope the revised wording in the manuscript satisfies your concerns in giving clear information to the readers.
Lines 341-342: I couldn’t find the source of Land Use and Land Cover result, once it is not in Jackknife analysis or elsewhere. Please, make it clearer. Also, there was not % residual?

Re: Sorry, it was an error while labelling/naming the file. “Land use and Land cover” was mistakenly labelled as “vegetation”. We have now corrected the text and figures accordingly.
Table 2: The sum of Contribution) is 100.1, and Permutation importance is 100.2. Please, re-round.

Re: Corrected
Line 366: Figure 3. Please, indicate the figure as 3a, 3b, 3c... in the figure and in the manuscript. It was a waste of time to connect figures and the corresponding text.

Re: Corrected. Please refer to line numbers 364,373,376, and 378.
Line 374: Put your graphic in %, following the manuscript.

Re: Added. Now at line 373.
Line 375: You forgot to mention the temperature in the driest quarter.

Re: Added. Please refer to line numbers 373-374.
Line 380: “Probability of presence”: Considering the statistical meaning of "probability", is it correct in a strict sense? If you are not sure about this, please replace it.

Re: We decided to keep only “the logistic output” and removed the “probability of presence”. Please refer to line numbers 383-384.
Lines 388 and 389: Be nice to your reader and enter the data here (%).

Re: Added. Please refer to line numbers 392-393.
Line 392: Potential or current?

Re: This is the potential current distribution range predicted by the MaxEnt model.
Lines 446 and 448: Could you please mark those cases on the table? Also, standardize the decimal places, and double-check.

Re: Done. We corrected the table and added an explanation “Districts with boldface are expected to gain suitable habitats while districts with italicized are expected to lose suitable habitats under the future climate scenarios” in the table legends. Please refer to line numbers 462-463.

Line 451: Table 4: Same as Line 296.

Re: Corrected

Line 460: Under 15 what?

Re: Corrected. The new wording in the revised manuscript appears as “Compared to current climatic conditions, the actual suitable area of *R. australe* is expected to decrease under all the future climate scenarios/ periods except for scenario SSP245 from 2081 to 2100 in which the suitable area of *R. australe* is expected to increase by 3.67% (Table 5)”. Please refer to line numbers 469-472

Line 492: Aspect: I suggest changing this term, since "aspect" can mean anything when alone.

Re: Corrected as “land aspect”.
Lines 506 and 507: It's okay to use Maxent, you don't have to justify yourself too much. But it's important to consider and make clear to the reader the limitations of this choice.

Re: We have added the limitation of the use of maxent and justification for the selection of Maxent model at line number 522-525 and 105-110.
Lines 509 and 511: Double-check the sentence, it seems weird.

Re: Thank you for pinpointing this weird sentence. We found that the information in it is redundant, and thus, it no longer appears in the revised manuscript.
Line 535: You spent a lot of time comparing your work with the work of other authors who talk about species other than yours. It seems quite clear to me that each species will respond differently to environmental variables. I suggest you rethink your approach. Suggestion: Explore the fact that environmental factors, such as elevation (not temperature), are so important to the species. What does this tell us?

Re: Considering the suggestion by reviewer 1 to add more examples to discuss the findings, we feel that some examples of previous works on the impact of climate change in the distribution pattern of medicinal plants in the mountainous regions would help to contextualize the findings. However, we have revisited the wording.

We have discussed the possible implications of the important role of elevation and precipitation-related factors (not temperature) in the distribution of our study species at line numbers 555-576.

Line 538: You say "studies", but you only point to one reference.

Re: Some more studies were added to justify this statement. Please refer to line number 578.

Lines 541-542: Why?

Re: Previous studies have suggested that short-distance assisted range expansion, i.e., the movement of a species to a higher place than it would normally occur but within its range limit, is the easiest strategy of a plant species to cope with the adverse effect of climate change (Casaza et al 2020, Ste-Marie, 2011). Consistent with these previous findings, several other herbaceous medicinal plants of the Himalayas are found migrating upward to mitigate the impact of climate change. Therefore, we speculated on the upward migration of the study species. We have added the explanation for this expectation in the manuscript at line numbers 577-586.

Lines 552-554: So... ? Why this is important? Did they use other approach, for example?

Re: We mean for other Rheum species and other medicinal plants in the Nepalese Himalaya. Text corrected accordingly. Please refer to line numbers 595-598.

Line 556: The fact that you have a general shrinkage of the suitable area and an increase in these areas in certain locations, does not mean that your results are inconsistent. Please choose a better word.

Re: We replaced the word “inconsistent” with “idiosyncratic”. Please refer to line number 631.
Lines 561-563: This is a nice result!!

Re: Thank you for your appreciation!
Line 570: And what does your models say?

Re: This is the prediction of our model.
Line 578: replace “;” to “,”.

Re: corrected
Line 582: What characterizes these scenarios?

Re: Specific characteristics of those scenarios are added in the manuscript. Please refer to 605-610
Lines 583-584: Are high emissions or their consequences limiting the occurrence of the species?

Re: Yes, corrected! Please refer to line number 611.
Lines 587-592: But here, in the Discussion section, you must discuss this result, not just let it go until another study comes along.

Re: We have revisited this paragraph.Please refer to line number 630-651
Lines 598-599: You should discuss this; not just say you don't know. It's okay to recognize the limitations of the model, but that doesn't prevent you from discussing the results based on the SDM science that exists to date.

Re: We have revisited this paragraph, and the previous sentences no longer appear in the manuscript. The revised paragraph is now at line numbers 500-554.

Lines 619-621: Please, rewrite.

Re: Corrected. Please refer to line numbers 707-708.
Line 634: Here you are making recommendations based on your findings. It is fine, but now you see how this recommendations are incongruous if you think about you tell us in the last section?

Re: We have eliminated our ambiguity about the model's result based on the existing SDM science.

6. PLOS authors have the option to publish the peer review history of their article ([what does this mean?](https://journals.plos.org/plosone/s/editorial-and-peer-review-process#loc-peer-review-history)). If published, this will include your full peer review and any attached files.


If you choose “no”, your identity will remain anonymous but your review may still be made public.


**Do you want your identity to be public for this peer review?** For information about this choice, including consent withdrawal, please see our [Privacy Policy](https://www.plos.org/privacy-policy).

Reviewer #1: No

Reviewer #2: No

[NOTE: If reviewer comments were submitted as an attachment file, they will be attached to this email and accessible via the submission site. Please log into your account, locate the manuscript record, and check for the action link "View Attachments". If this link does not appear, there are no attachment files.]

While revising your submission, please upload your figure files to the Preflight Analysis and Conversion Engine (PACE) digital diagnostic tool, <https://pacev2.apexcovantage.com/>. PACE helps ensure that figures meet PLOS requirements. To use PACE, you must first register as a user. Registration is free. Then, login and navigate to the UPLOAD tab, where you will find detailed instructions on how to use the tool. If you encounter any issues or have any questions when using PACE, please email PLOS at [figures@plos.org](mailto:figures@plos.org). Please note that Supporting Information files do not need this step.
